# Identification of environment-insensitive genes for oil content by combination of transcriptome and genome-wide association analysis in rapeseed

**DOI:** 10.1186/s13068-024-02480-x

**Published:** 2024-02-22

**Authors:** Min Yao, Dan He, Wen Li, Xinghua Xiong, Xin He, Zhongsong Liu, Chunyun Guan, Lunwen Qian

**Affiliations:** 1 College of Agronomy, Hunan Agricultural University, Changsha, 410128 China; 2 Yuelushan Laboratory, Changsha, 410128 China

**Keywords:** GWAS, Haplotype, Seed oil content, Co-expression, Environment-insensitive, *Brassica napus*

## Abstract

**Background:**

The primary objective of rapeseed breeding is to enhance oil content, which is predominantly influenced by environmental factors. However, the molecular mechanisms underlying the impact of these environmental factors on oil accumulation remain inadequately elucidated. In this study, we used transcriptome data from two higher (HOC) and two lower oil content (LOC) inbred lines at 35 days after pollination (DAP) to investigate genes exhibiting stable expression across three different environments. Meanwhile, a genome-wide association study (GWAS) was utilized to detect candidate genes exhibiting significant associations with seed oil content across three distinct environments.

**Results:**

The study found a total of 405 stable differentially expressed genes (DEGs), including 25 involved in lipid/fatty acid metabolism and 14 classified as transcription factors. Among these genes, *BnBZIP10-*A09, *BnMYB61*-A06, *BnAPA1*-A08, *BnPAS2*-A10, *BnLCAT3*-C05 and *BnKASIII*-C09 were also found to exhibit significant associations with oil content across multiple different environments based on GWAS of 50 re-sequenced semi-winter rapeseed inbred lines and previously reported intervals. Otherwise, we revealed the presence of additive effects among *BnBZIP10-*A09, *BnKASIII*-C09, *BnPAS2*-A10 and *BnAPA1*-A08, resulting in a significant increase in seed oil content. Meanwhile, the majority of these stable DEGs are interconnected either directly or indirectly through co-expression network analysis, thereby giving rise to an elaborate molecular network implicated in the potential regulation of seed oil accumulation and stability.

**Conclusions:**

The combination of transcription and GWAS revealed that natural variation in six environment-insensitive gene regions exhibited significant correlations with seed oil content phenotypes. These results provide important molecular marker information for us to further improve oil content accumulation and stability in rapeseed.

**Supplementary Information:**

The online version contains supplementary material available at 10.1186/s13068-024-02480-x.

## Introduction

With the development of society, the consumption demands for edible oil are increasing rapidly. The majority of vegetable oils are derived from four major crops, namely soybean, rapeseed, oil palm, and sunflower [1]. The global supply of edible oil is boosted by rapeseed, accounting for over 15% [2]. The cultivation of rapeseed plays a pivotal role in providing essential edible oil in the European Union, Canada, and China. The increasing demand for edible oil among consumers necessitates the imperative to enhance both seed oil content and oil production per unit area of land in rapeseed breeding.

The oil content of seeds is highly susceptible to environmental factors and exhibits variations ranging from 35 to 55% across diverse ecological zones and climatic conditions [3]. Low temperature increases the polyunsaturated fatty acid content in plants, thereby contributing to the maintenance of biological membrane fluidity [4]. Bellaloui et al. demonstrated that elevated temperatures had an impact on both the production and composition of oil, potentially due in part to the restricted availability and transport of carbohydrates from leaves to seeds [5]. Zhou et al. suggested that temperature causes changes in the expression of lipid metabolism genes, thereby regulating lipid accumulation in rapeseed [3]. The expression stability of the *FAD2* and *FAD3* genes during lipid accumulation may be directly influenced by temperature, as suggested by some research [6–8]. Meanwhile, light intensity is an essential factor in determining photosynthetic efficiency. Light intensity affects gene expression in lipid metabolism, which regulates the seed oil content in developing seeds [9, 10]. The expression of *WRINKLED1*, a crucial gene involved in regulating lipid biosynthesis, is closely linked to the photosynthetic activity of the silique wall during seed development [11]. Although the molecular mechanisms underlying the regulation of lipid/fatty acid accumulation by temperature and light remain poorly understood, identifying genes involved in lipid/fatty acid metabolism that are insensitive to environmental factors may offer a more effective approach to improve oil content stability and optimize fatty acid composition.

Environmental and genotypic interactions lead to gene expression pattern differences that result in phenotypic diversity. With the next-generation sequencing development, gene expression variation can be measured quantitatively, and DEGs related to phenotypes and/or environments can be explored. The application of transcriptome analysis has been instrumental in unraveling the DEGs implicated in modulating the oil content of rapeseed [12, 13]. Numerous DEGs could be identified in short times by this application. However, the transcriptome was only interpreted as phenotypic variation in terms of gene expression and failed to fully interpret genetic variations [14]. Meanwhile, numerous differentially expressed genes make explaining transcriptome results and enacting breeding strategies more difficult. GWAS is an application that studies complex phenotypes by investigating genetic variations in the whole genome and has been extensively applied in rapeseed [15–17]. Therefore, combining transcriptome analysis and GWAS to identify differentially expressed genes and explore genetic variations is a novel strategy. For example, Zhang et al. used GWAS combined with transcriptome analysis to reveal that *HCTs* and *WRKYs* interact to regulate the defence response of poplar [18]. Xiao et al. identified a few key genes of the lipid biosynthesis pathway controlling oil content by combining GWAS and transcriptome analysis in rapeseed [19]. The combination of GWAS and transcriptome analysis was employed to detect ten candidate genes implicated in the regulation of flax seed fatty acid metabolism in rapeseed [20].

In this study, we carried out transcriptome sequencing of two HOC and LOC accessions at 35 DAP across three different environments. Meanwhile, a GWAS was conducted to identify candidate genes that demonstrate significant associations with seed oil content across three distinct environmental conditions. Our aim was to identify environment-insensitive genes in the process of oil accumulation. The research results will provide theoretical basis for breeding varieties with high and stable oil content in rapeseed.

## Results

### Changes in oil content in developmental stages and different environments

**Fig. 1:**
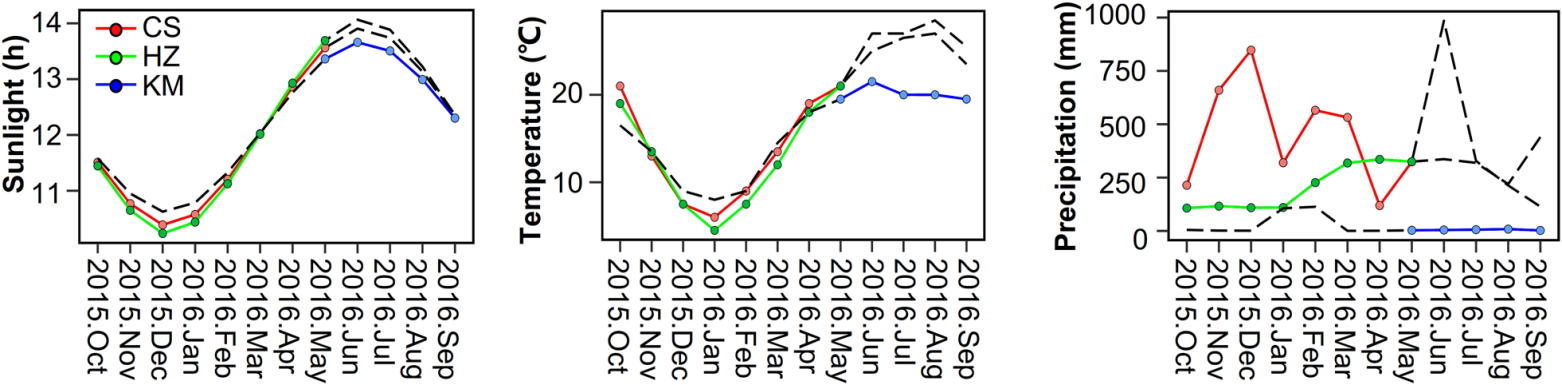
Climate data for three environments tested. The graphs show sunlight (monthly mean values), temperature (monthly mean values), and precipitation (monthly sums) for each environment. The environments are abbreviated as follows: Changsha (CS), Hangzhou (HZ) and Kunming (KM) in China

To investigate the dynamics of oil accumulation during seed development, we measured the seed oil content of two HOC and LOC accessions at 20, 25, 30, 35, 40, and 45 DAP under three distinct environmental conditions. CS and HZ are characterized by low temperatures and high levels of precipitation, in contrast to KM (Fig. 1). The seed oil content accumulation in CS and HZ occurred between 20 and 40 DAP, with the period from 30 to 35 DAP exhibiting the most rapid increase, followed by a decline observed at 40 to 45 DAP. While the seed oil content at 20–35 DAP showed a faster increase, the period at 35–45 DAP exhibited a downward increase in KM (Additional file 1: Fig. S1a). HOC and LOC reached significant differences at 35 DAP in the three different environments (Additional file 1: Fig. S1a). All genes expression patterns of HOC and LOC at 35 DAP across the three environments were characterized through a principal component analysis (PCA). The PC1 explained 14.0% of the variation and exhibited distinct separation between the HOC and LOC groups (Additional file 1: Fig. S1b). These results indicate that gene expression is more stable in different environments at 35 DAP. Therefore, we particularly aimed at transcriptome data from 35 DAP to explore the stably expression genes involved in the process of lipid accumulation in different environments.

### Identification of environment-insensitive genes during the accumulation of oil

To investigate environment-insensitive genes associated with seed oil content, we analyzed the stable differentially expressed genes (DEGs) between two HOC and LOC accessions at 35 DAP in three different environments: CS, HZ, and KM. The DEGs were defined as those with a fold change of FPKM expression values that were at least 2 in either direction, when the *q* value or FDR < 0.001 and |Log_2_ fold change| ≥ 1.

**Fig. 2:**
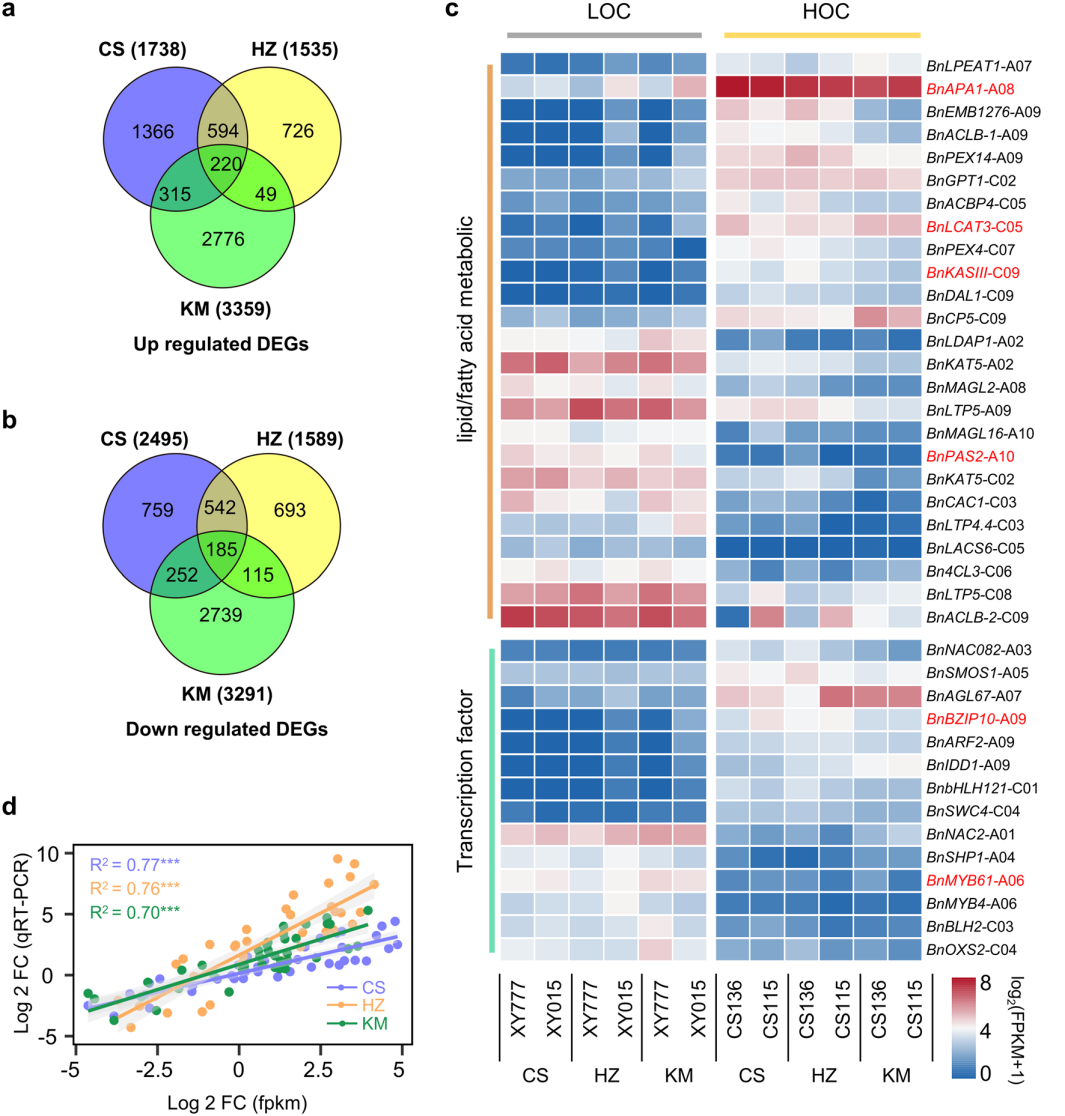
Summary of DEGs between the extremely HOC and LOC inbred lines. Number of stable up-regulated (**a**) and down-regulated (**b**) DEGs in CS, HZ and KM. **c** The heatmap of 39 selected stable DEGs from the comparison between HOC and LOC inbred lines in three different environments. Three biological replicates were used to calculate expression values with three technical replicates, and normalized by log_10_^(mean expression values)^. **d** Transcriptome sequencing verified by qRT-PCR. Normalized expression (2^−∆∆Ct^) in HOC inbred lines was divided by normalized expression in LOC inbred lines and log^2 transformed^. The correlation coefficient (*R*^2^) was calculated for comparison. The orange, green and turquoise lines represent the CS, HZ and KM environment fitted curves, respectively. *p* values show the significance of pairwise comparisons

We identified 1738, 1535, and 3359 up-regulated genes in two HOCs compared with two LOCs under CS, HZ, and KM, respectively, and 2495, 1589, and 3291 down-regulated genes were discovered, respectively (Additional file 2: Fig. S2b). As illustrated in Fig. 2a, 220 and 185 genes were stably up- and down-regulated in the three environments, respectively, indicating that these genes were environment-insensitive genes. The KEGG enrichment analysis revealed a significant enrichment of these 405 stable DEGs in various pathways, including flavonoid biosynthesis, lipid metabolism, energy metabolism, and more (Additional file 2: Fig. S2c). Among them, we found 25 stable DEGs involved in fatty acid/lipid metabolism, and 14 stable DEGs were transcription factors (Fig. 2c, Additional file 7: Table S1). Next, these 39 stable DEGs were selected to evaluate the accuracy of RNA‒Seq by qRT‒PCR analysis. The expression patterns of these stable DEGs by RNA-Seq and qRT‒PCR that exhibited a high degree of correlation (*R*^2^ = 0.70–0.77), thereby providing further validation and consistency with the RNA sequencing data (Fig. 2d).

**Fig. 3:**
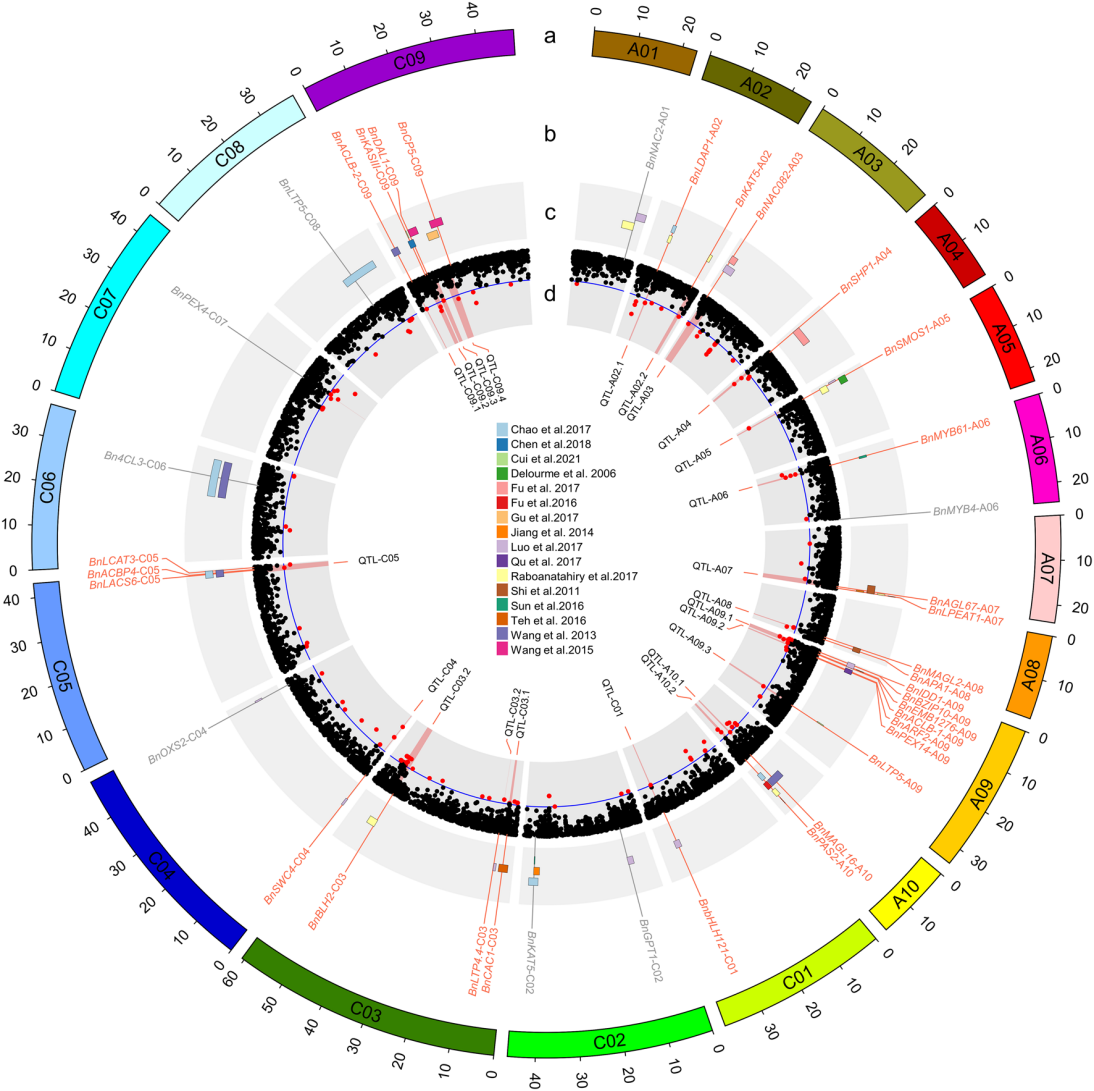
The circos plot of 39 selected stable DEGs, previously reported QTLs regions and GWAS for oil content. **a** Chromosomes; **b** position of 39 stable DEGs, the red font represents genes that are located in the overlapping interval between reported interval and the significantly associated haplotype regions; **c** QTLs and association region genetic intervals from published studies; **d** GWAS of seed oil content, the blue line represents −log_10_^*p*−value^ ≥ 4.0. Red fill represents significant QTLs

GWAS was employed to detect associations between SNPs and oil content in 50 semi-winter rapeseed inbred lines. The Manhattan plots in Fig. 3d depict numerous significant SNP associations with seed oil content observed in glasshouse and field experiments. The study identified 35 haplotype (Hap) regions that exhibited a significant association with seed oil content, including 23 regions that overlapped with previously reported intervals (Fig. 3c and d; Additional file 8: Table S2). The combination of the aforementioned 39 stable DEGs revealed that 31 of them were situated within these overlapping regions (Fig. 3b; Additional file 8: Table S2).

**Fig. 4:**
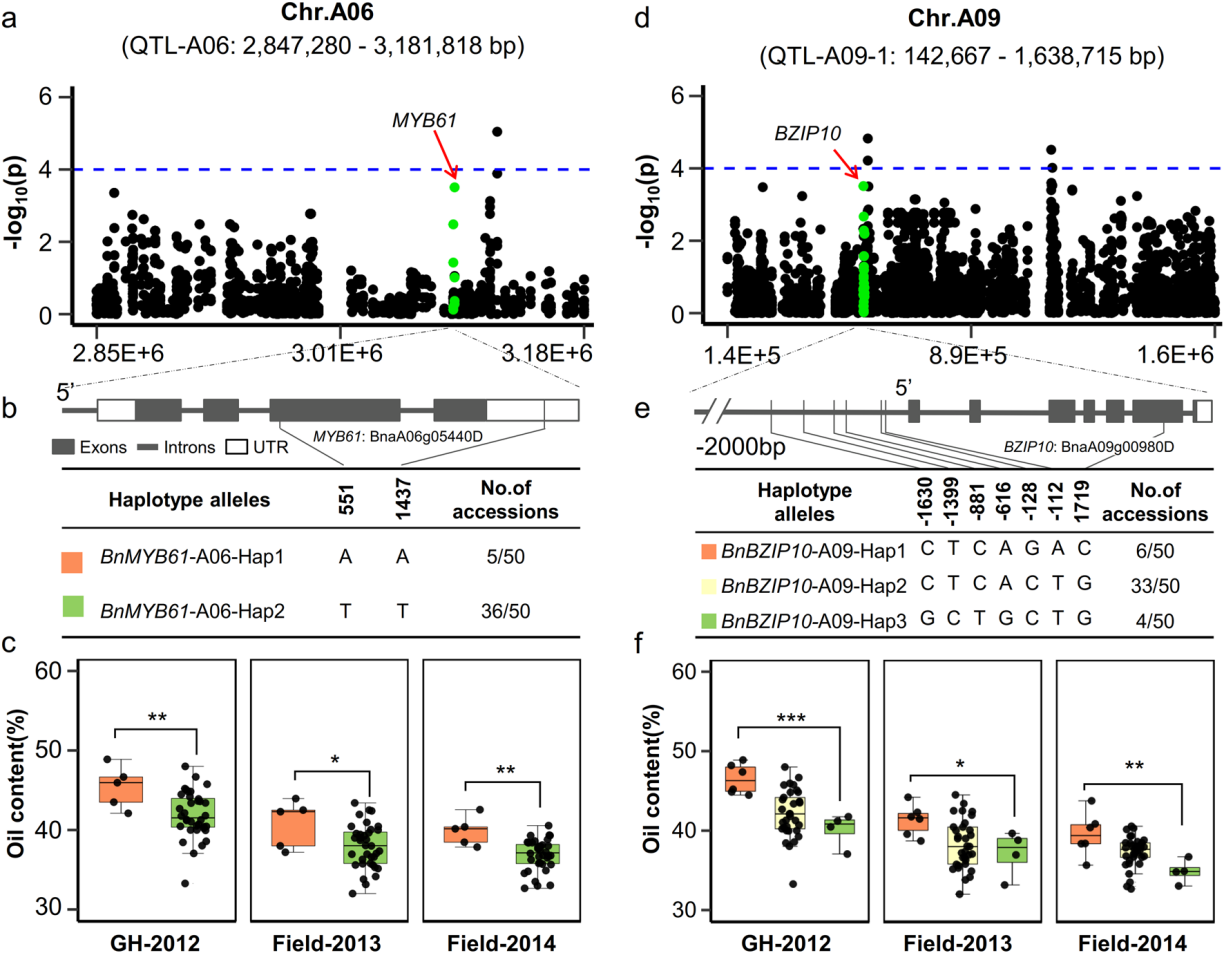
Analysis of candidate genes in the significantly associated QTL-A06 and QTL-A09 regions. Regional Manhattan plot surrounding the peak signals on QTL-A06 (**a**) and QTL-A09 (**d**). Green dots indicate SNPs located in the *BnMYB61*-A06 (**a**) and *BnBZIP10*-A09 (**d**) gene regions which significantly associated with oil content. Genetic structure variations of *BnMYB61*-A06 (**b**) and *BnBZIP10*-A09 (**e**). **c**, **f** Boxplots showing comparative analysis between haplotypes related to the oil content phenotype. *p* values show the significance of pairwise comparisons

Of these 31 DEGs, two transcription factors *BnMYB61*-A06 and *BnBZIP10-*A09 were located in QTL-A06 (2,847,280–3,181,818 bp) and QTL-A09.1 (142,667–1,638,715 bp), respectively (Fig. 4). Two and three haplotype alleles were identified within these two gene regions, and *BnMYB61*-A06-Hap1 and *BnBZIP10-*A09-Hap1 corresponded to inbred lines exhibiting relatively high oil content (Fig. 4c and f; Additional file 9: Table S3). Four lipid/fatty acid synthesis genes, namely, *BnAPA1*-A08, *BnPAS2*-A10, *BnLCAT3*-C05 and *BnKASIII*-C09 located within QTL-A08 (16,454,214–17,628,998 bp), QTL-A10 (14,388,914–15,045,397 bp), QTL-C05 (41,242,900–42,812,067 bp), and QTL-C09.2 (6,631,263–7,574,558 bp), respectively (Additional file 3: Fig. S3 and Additional file 4: Fig. S4). Two, four, two, and three haplotype alleles were found in these four gene regions, and *BnAPA1*-A08-Hap1, *BnPAS2*-A10-Hap1, *BnLCAT3*-C05-Hap1, and *BnKASIII*-C09-Hap1 had higher oil contents than the other haplotype alleles (Additional file 3: Fig. S3c and f; Additional file 4: Fig. S4c and f; Additional file 9: Table S3).

### Additive effects analysis of environment-insensitive genes

**Fig. 5:**
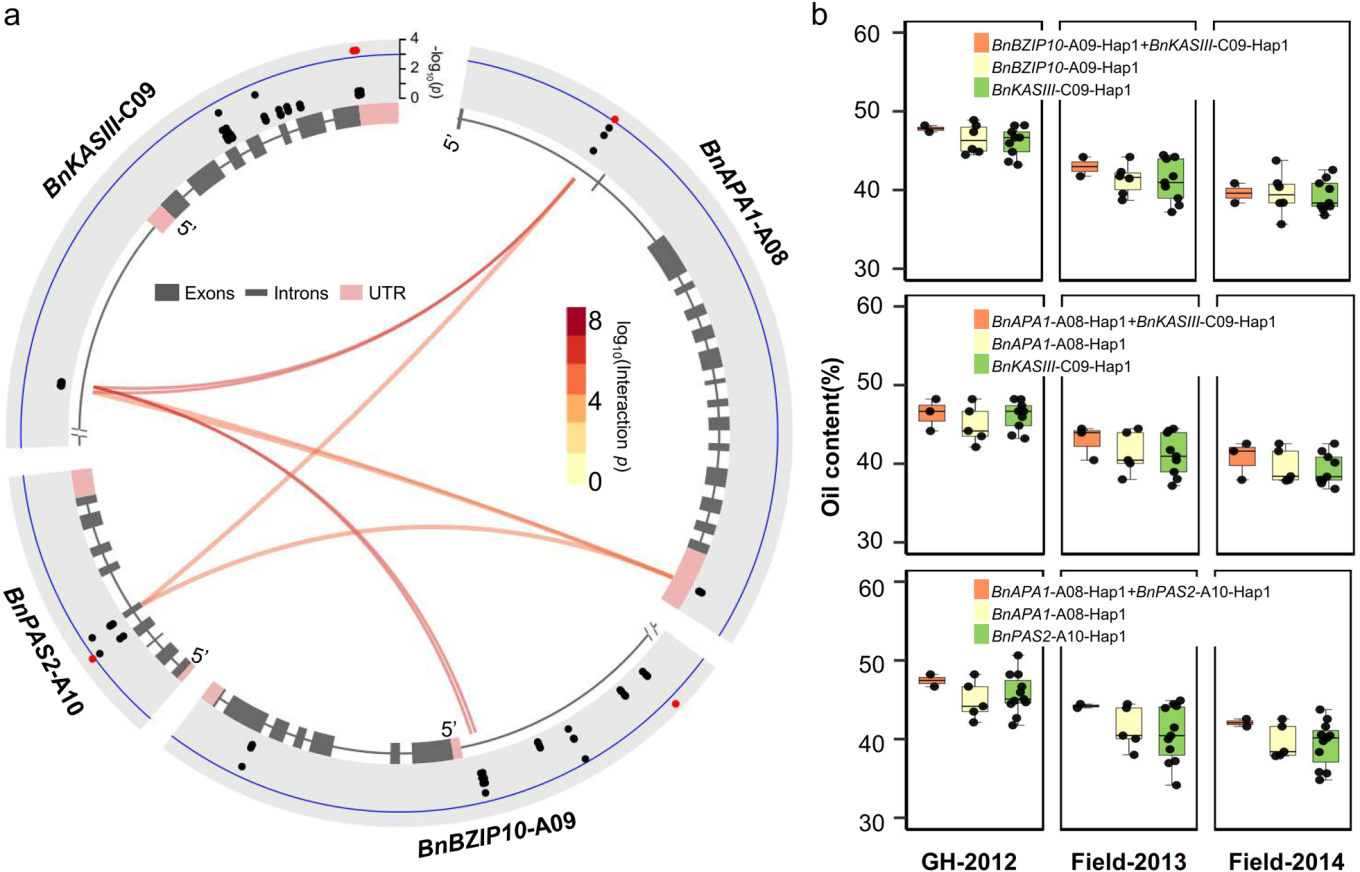
Additive effect analysis between candidate genes. **a** Circos plot showing candidate gene interaction analysis by the R package “SIPI”. The dots in the outer arcs represent −log_10_^(*p*−value)^ of GWAS, and the secondary arcs represent the candidate gene structure. The links positioned at the center of the circle symbolize the additive-by-additive relationship between two SNPs. **b** Boxplots showing the oil content of additive-by-additive haplotypes which is higher than other of a single haplotype.* p* values show the significance of pairwise comparisons

To perform a single-variant-additive-effect analysis in GWAS, we investigated the interaction effects on oil content among both significant and non-significant SNP markers within these six candidate gene regions. Our findings revealed that the combination of *BnKASIII*-C09 and *BnAPA1*-A08, *BnAPA1*-A08 and *BnPAS2*-A10, as well as *BnKASIII*-C09 and *BnBZIP10*-A09, exhibited an additive effect (Fig. 5a, Additional file 12: Table S6). Additionally, we identified three combinations of haplotype alleles (*BnKASIII*-C09-Hap1 + *BnAPA1*-A08-Hap1, *BnKASIII*-C09-Hap1 + *BnBZIP10*-A09-Hap1, *BnAPA1*-A08-Hap1 + *BnPAS2*-A10-Hap1) that correspond to inbred lines exhibiting relatively high oil content compared to single haplotype allele (*BnKASIII*-C09-Hap1, *BnAPA1*-A08-Hap1, *BnBZIP10*-A09-Hap1, and *BnPAS2*-A10-Hap1) (Fig. 5b). These results suggest potential additive effects of these four genes on oil content.

### Co-expression network analysis of environment-insensitive genes

To provide additional context for the proposed functions of *BnKASIII-C09*, *BnBZIP10*-A09, *BnAPA1*-A08, *BnLCAT3*-C05, Bn*PAS2*-A10, and *BnMYB61*-A06, we utilized transcriptome data from four accessions across three different environments at 35 DAP to construct intricate co-expression networks. This analysis resulted in the identification of 12 gene modules. The *BnKASIII-C09*, *BnBZIP10*-A09, *BnAPA1*-A08, and *BnLCAT3*-C05 genes fell in the blue module, which showed a significant positive correlation with oil content (*r* 0.84; Additional file 5: Fig. S5b). The turquoise module including the *BnPAS2*-A10 and *BnMYB61*-A06 genes showed a significant positive correlation with oil content (*r* 0.53; Additional file 5: Fig. S5b, Additional file 6: Fig. S6a). KEGG enrichment analysis of module genes was performed. The module genes were subjected to KEGG enrichment analysis. The blue module exhibited significant enrichment in unsaturated fatty acid biosynthesis, fatty acid degradation, and flavonoid biosynthesis, whereas the turquoise module showed significant enrichment in fatty acid biosynthesis, flavonoid biosynthesis, and photosynthesis (Additional file 6: Fig. S6b).

**Fig. 6:**
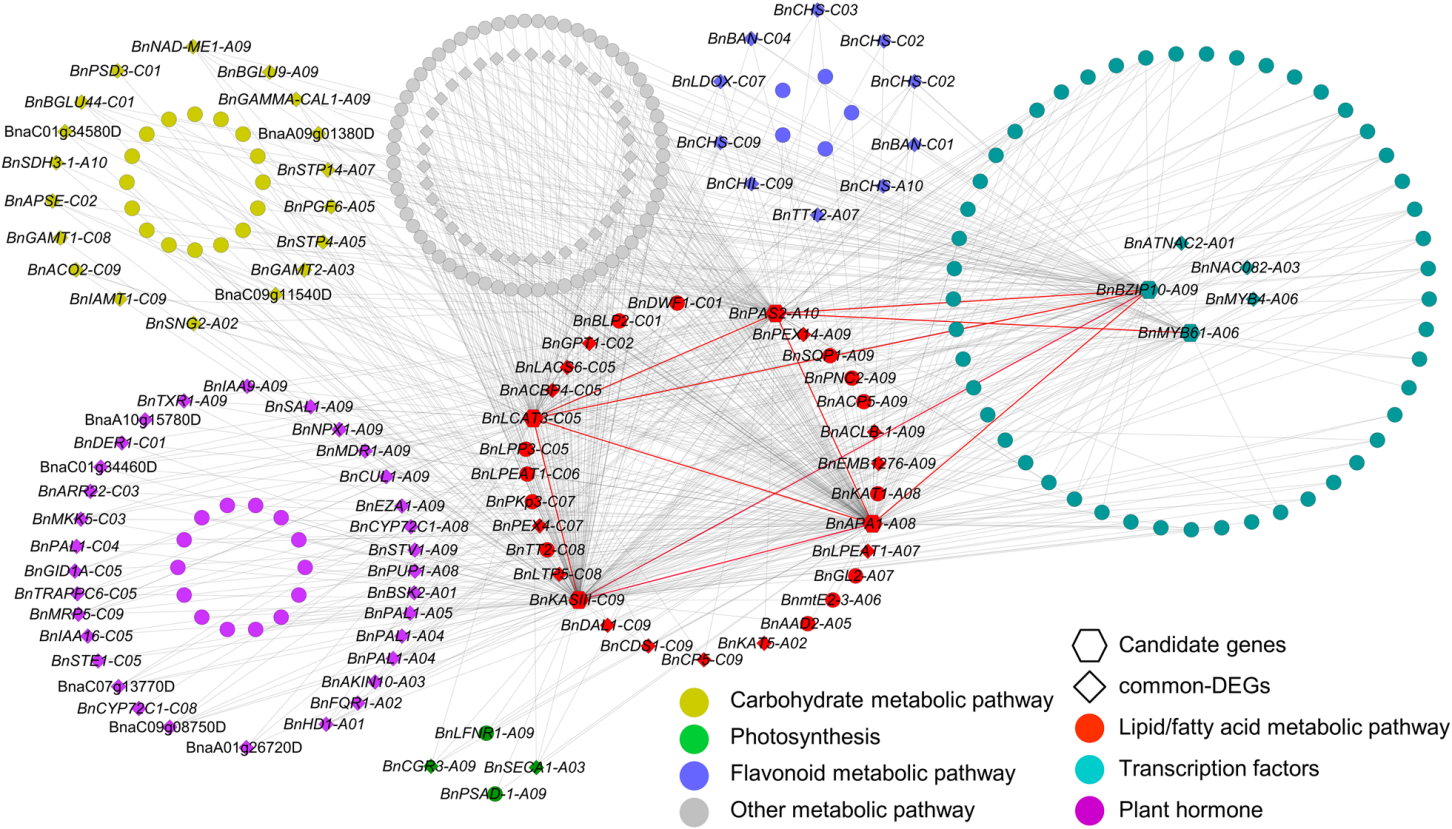
Co-expression network analysis. Hexagon nodes represent eight candidate genes, and rhombus nodes represent common DEGs in three environments. Co-expression network into the following groups based on functional annotation: red nodes representing lipid/fatty acid biosynthetic process, turquoise nodes representing transcription factors, wathet blue nodes representing flavonoid metabolic pathway, brown nodes representing carbohydrate metabolic pathway, green nodes representing photosynthesis, and purple nodes representing plant hormones

The subnetwork contained a total of 286 genes, including 125 stable DEGs (Fig. 6, Additional file 10: Table S4). The set of stable DEGs consisted of 30, 34, 15, and 46 genes involved in fatty acid/lipid metabolic processes, carbohydrate metabolic processes, flavonoid metabolism pathways, and plant hormone metabolic pathways, respectively. Further analysis of candidate gene subnetworks revealed that *BnBZIP10*-A09 is directly linked to *BnKASIII*-C09, *BnAPA1*-A08, *BnLCAT3*-C05, and *BnPAS2*-A10, while *BnMYB61*-A06 is directly linked to *BnPAS2*-A10 (Fig. 6). These results suggest that these genes establish a potential molecular network that impacts the accumulation and stability of oil content in rapeseed.

## Discussion

The augmentation of seed oil content stands as a paramount objective in the realm of rapeseed breeding. The regulation of oil accumulation in seeds is governed by a complex network of genes and is highly susceptible to environmental influences [21–23]. To date, extensive investigations have been conducted on numerous QTLs associated with seed oil content in rapeseed, including some environment-insensitive QTLs identified across different environments [24–27]. Building on previous research findings, environment-insensitive genes might exist and regulate oil content accumulation in rapeseed.

In this study, we initially investigated the dynamics of oil accumulation at six different developmental stages (20, 25, 30, 35, 40, and 45 DAP) under three distinct environmental conditions. Our findings revealed key stages of oil synthesis occurring between 30 and 35 DAP, which aligns with the findings reported in previous studies [12]. To identify genes that show insensitivity to environmental changes, we conducted a transcriptome analysis of HOC and LOC inbred lines at 35 DAP across three distinct environments. As a result, we discovered 25 stable DEGs involved in lipid/fatty acid metabolism, while 14 stable DEGs were identified as transcription factors (Fig. 2c), indicating that these genes are environment-insensitive and associated with the oil content accumulation of rapeseed.

Meanwhile, *BnAPA1*-A08, *BnPAS2*-A10, *BnLCAT3*-C05, *BnKASIII*-C09, *BnMYB61*-A06, and *BnBZIP10-*A09 were identified as environment-insensitive genes affecting the oil content accumulation in rapeseed by transcriptome analysis and GWAS. *APA1* and *LCAT3* are involved in lipid metabolic processes [28, 29]. The interaction between *PAS2* and *CER10* suggests their participation in very long-chain fatty acid biosynthesis [30]. Dehesh et al. suggested that *BnKASIII* affects oil content accumulation in rapeseed [31]. *BZIP10* forms a ternary complex with *BZIP53* and *ABI3* to promote the expression of seed maturation genes and affect seed oil accumulation [32–34]. The positive regulation of *MYB61* on *GL2* exerts a partial inhibition the accumulation of oil content, partly by modulating the formation of mucilage in the seed coat [35, 36]. Moreover, the co-expression network revealed that *BnBZIP10*-A09 is directly linked to *BnKASIII*-C09, *BnAPA1*-A08, *BnLCAT3*-C05, and *BnPAS2*-A10. *BnMYB61*-A06 is directly linked to *BnPAS2*-A10 and *BnGL2*-A07. The results suggest that fatty acid/lipid metabolism and transcription factors potentially interact and play a role in regulating the accumulation and stability of seed oil content in rapeseed.

The constant updates in sequencing technology and the continuous decrease in sequencing costs have stimulated large-scale germplasm sequencing projects in crops, thereby creating exciting opportunities for utilizing haplotypes in breeding applications. The observed haplotype alleles had a significantly higher level of diversity compared to the typical intra-species variation, indicating that successful reconstruction of haplotypes in polyploid species will have a substantial impact on crop breeding in the future [37]. Recently, researchers have utilized large-scale population sequencing data to detect favorable haplotype alleles associated with enhanced cold tolerance in rice [38], drought tolerance in maize [39], and head blight resistance in wheat [40]. In our study, we identified six favorable haplotype alleles corresponding to inbred lines exhibiting relatively high oil content, and three of these alleles had additive effects with each other. Voss-Fels et al. revealed the presence of an additive × additive epistasis effect between the two haplotypes, resulting in a significant increase in root biomass in wheat [41]. An additive effect was found between the two haplotypes, which significantly increased the chlorophyll content [42]. Our results will provide favorable haplotype alleles for further enhancement of seed oil content accumulation and stability in rapeseed.

## Conclusions

The combination of transcription and GWAS revealed that natural variation in six environment-insensitive gene regions (*BnBZIP10*-A09, *BnMYB61*-A06, *BnAPA1*-A08, *BnPAS2*-A10, *BnLCAT3*-C05, and *BnKASIII*-C09) correlated with the seed oil content phenotypic variation. Additionally, we also found the presence of additive effects among *BnBZIP10-*A09, *BnKASIII*-C09, and *BnAPA1*-A08, resulting in a significant increase in seed oil content. Meanwhile, co-expression network analysis revealed that most of these stable DEGs are interconnected either directly or indirectly, thereby forming a molecular network implicated in the potential regulation of the concentration accumulation and stability of seed oil in rapeseed. The findings will offer valuable molecular markers for enhancing the high and stable oil content varieties in *B.napus*.

## Materials and methods

### Plant materials

Four rapeseed inbred lines, XY777 and XY015 (LOC) and CS136 and CS511 (HOC), came from Hunan Agricultural University, China, and were planted in Changsha (CS; E112.938888, N28.228272), Hangzhou (HZ; E120.15358, N30.287458) and Kunming (KM; E102.71225, N25.040609) in China. These four accessions were sown on October 10, 2015, in CS and HZ and in KM on May 15, 2016. Seed tissues were sampled with three biological replicates at 20, 25, 30, 35, 40, and 45 DAP. To measure the seed oil content using the Soxhlet extraction method to determine fat in foods (GB 5009.6-2016, standardization administration, China). The origin and oil content phenotypes of 50 Chinese semi-winter rapeseed inbred lines have been described in detail by Yao et al. [43].

### Gene expression analysis

A detailed description of transcriptome sequencing in four inbred lines has been provided by Jia et al. [44]. The R package DESeq2 v1.24.0 [45] was employed to determine the differential expression genes when comparing each HOC with each LOC under CS, HZ, and KM, respectively, *q* value < 0.001 and |Log_2_ fold change| ≥ 1 were defined as differential expression genes. DEGs that were up- or down-regulated in all three environments were defined as environment-insensitive genes. Gene sequences from the *B. napus* reference genome (http://www.genoscope.cns.fr/brassicanapus/) blast to the *Arabidopsis* genome database (http://www.arabidopsis.org/) were used to assign putative gene functions.

KEGG enrichment analysis was conducted by TBtools [46]. The results were visualized using “ggplot2” [47].

### Real-Time Quantitative PCR verification

cDNAs were synthesized using the same RNAs as those used for RNA-seq. The analysis of the results was conducted using LightCycler 480 SYBR Green I Mastermix and a LightCycler 480II real-time PCR system (Roche, Switzerland). The expression pattern of all selected stable DEGs was verified by qRT-PCR using gene copy-specific primers, and data were normalized by *BnEF* [48] independently (primer showen in Additional file 11: Table S5). The 2^−ΔΔCT^ method [49] was used to access the fold change. The relative expression quantified by qRT‒PCR was converted to log2-fold change for direct comparison with RNA-Seq data.

### Genome-wide association analysis

The resequencing of 50 rapeseed inbred lines and the acquisition of 532,005 high-quality SNPs were comprehensively described by Dong et al. [50]. TASSEL 5.0 software was used to perform the relative kinship and PCA [51]. Based on PCA and relative kinship (P and K matrix), the calculation was performed with a mixed linear model (MLM) incorporated into TASSEL 5.0 software. The critical *p* value for assessing the significance of SNP-trait associations was calculated for seed oil content based on the false discovery rate (FDR) [52]. An FDR < 0.05 was used to identify significant marker‒trait associations for oil content at *P* value threshold of 1 × 10^−4^.

### Additive effect analysis

The R package “SIPI” [53] was used to evaluate pairwise interactions between the candidate gene SNP markers. In this study, only additive–additive interactions were considered. Wald *p* values < 0.01 were defined as significant SNP pairs.

### Weighted gene co-expression network analysis (WGCNA) for candidate genes

A total of 14,019 genes had FPKM max and a mean value greater than 10 and 2, respectively. The module identification was implemented by merging modules with similar expression profiles using a merge Cut Height of 0.25. The co-expression network was constructed by the R package WGCNA v1.69 [54] and visualized through Cytoscape 3.5.1 [55].

### Supplementary Information

**Additional file 1: Figure S1.** Oil content phenotype of the development period and PCA distribution for LOC and HOC inbred lines using 35 DAPs FPKM. **a** The seed oil content of LOC and HOC inbred lines at different environments in different growth stage, the blue and gray, red and orange lines represent LOC and HOC inbred lines, respectively. **b** PCA distribution for LOC and HOC inbred lines using 35 DAPs FPKM. CS, HZ and KM was signed by cycle, tangle and rhombus, respectively.

**Additional file 2: Figure S2.** Overview of DEGs in 35 DAP seeds of the HOC compared to LOC inbred lines. **a** DEG number of different comparisons at 35 DAP seed. **b** DEGs overlapped in the same environment under study. **c** Top 20 KEGG enhancement of common DEGs in three environments.

**Additional file 3: Figure S3.** The analysis of candidate genes in the significant associated QTL-A08 and QTL-A10 regions. Regional Manhattan plot surrounding the peak signals on QTL-A08 (**a**) and QTL-A10 (**d**). Green dot indicates the SNPs located in the *BnAPA1*-A08 (a) and *BnPAS2*-A10 (**d**) gene region which are associated with oil content. Genetic structure variations of *BnAPA1*-A08 (**b**) and *BnPAS2*-A10 (**e**). **c** and **f** Boxplots showing comparative analysis between haplotypes related to oil content phenotype.* p* values show the significance of pairwise comparisons.

**Additional file 4: Figure S4.** The analysis of candidate genes in the significant associated QTL-C05 and QTL- QTL-C09.2 regions. Regional Manhattan plot surrounding the peak signals on QTL-C05 (**a**) and QTL-C09.2 (**d**). Green dot indicates the SNPs located in *BnLCAT3*-C05 (**a**) and *BnKASIII*-C09 (**d**) which associated with oil content. Genetic structure variations of *BnLCAT3*-C05 (**b**) and *BnKASIII*-C09 (**e**), numbers indicate the SNP positions from gene start site. **c** and **f** Boxplots showed comparative analysis between haplotypes related to oil content phenotype.* p* values show the significance of pairwise comparisons.

**Additional file 5: Figure S5.** The result of co-expression network analysis. **a** Cluster dendrogram of WGCNA gene modules. **b** The information of module-trait coefficient and module gene numbers.

**Additional file 6: Figure S6.** Co-expression network analysis. **a** Whole co-expression network exhibit, hexagon nodes represent eight candidate genes, triangle nodes represent genes directly linked to the candidate gene. The blue and turquoise nodes represent blue and turquoise module genes. **b** Top 20 KEGG enhancement of blue and turquoise module genes.

**Additional file 7: Table S1.** The detailed information of 39 stable DEGs.

**Additional file 8: Table S2.** The information of chromosome positions of 50 stable DEGS, GWAS of oil content and previous reported QTL intervals.

**Additional file 9: Table S3.** Haplotype analysis of candidate genes region in 50 resequenced accessions.

**Additional file 10: Table S4.** Gene information in the co-expression network.

**Additional file 11: Table S5.** Primers information of qRT‒PCR.

**Additional file 12: Table S6.** The detailed information of additive affect between haplotypes in 50 resequenced accessions.

## Data Availability

The entirety of the data generated or analyzed throughout this study has been incorporated within this published article and its additional files.

## Abbreviations

GWAS: Genome-wide association study
DEGs: Differentially expressed genes
CS: Changsha
HZ: Hangzhou
KM: Kunming
SNP: Single nucleotide polymorphism
DAP: Days after pollination
QTL: Quantitative trait locus
HOC: High-oil content accessions
LOC: Low-oil content accessions
Hap: Haplotype
qRT‒PCR: Quantitative real-time PCR
*WRINKLED1*: WRINKLED 1
*FAD2*: Fatty acid desaturase 2
*FAD3*: Fatty acid desaturase 3
FPKM: The fragments per kilobase of transcript per million reads
PCR: Polymerase chain reaction
*KASIII*: 3-Ketoacyl-ACP synthase III
*BZIP10/53*: Basic leucine zipper 10/53
*APA1*: Aspartic proteinase A1
*LCAT3*: Lecithin: cholesterol acyltransferase 3
*PAS2*: PASTICCINO 2
*MYB61*: MYB domain protein 61
*CER10*: Eceriferum 10
*ABI3*: Abscisic acid insensitive 3
*GL2*: Glabra 2
