# Supplementary figures and images for: Identification of environment-insensitive genes for oil content by combination of transcriptome and genome-wide association analysis in rapeseed
Source: Biotechnol Biofuels Bioprod. 2024 Feb 22;17:29. doi: 10.1186/s13068-024-02480-x (PMC10882896; doi:10.1186/s13068-024-02480-x)

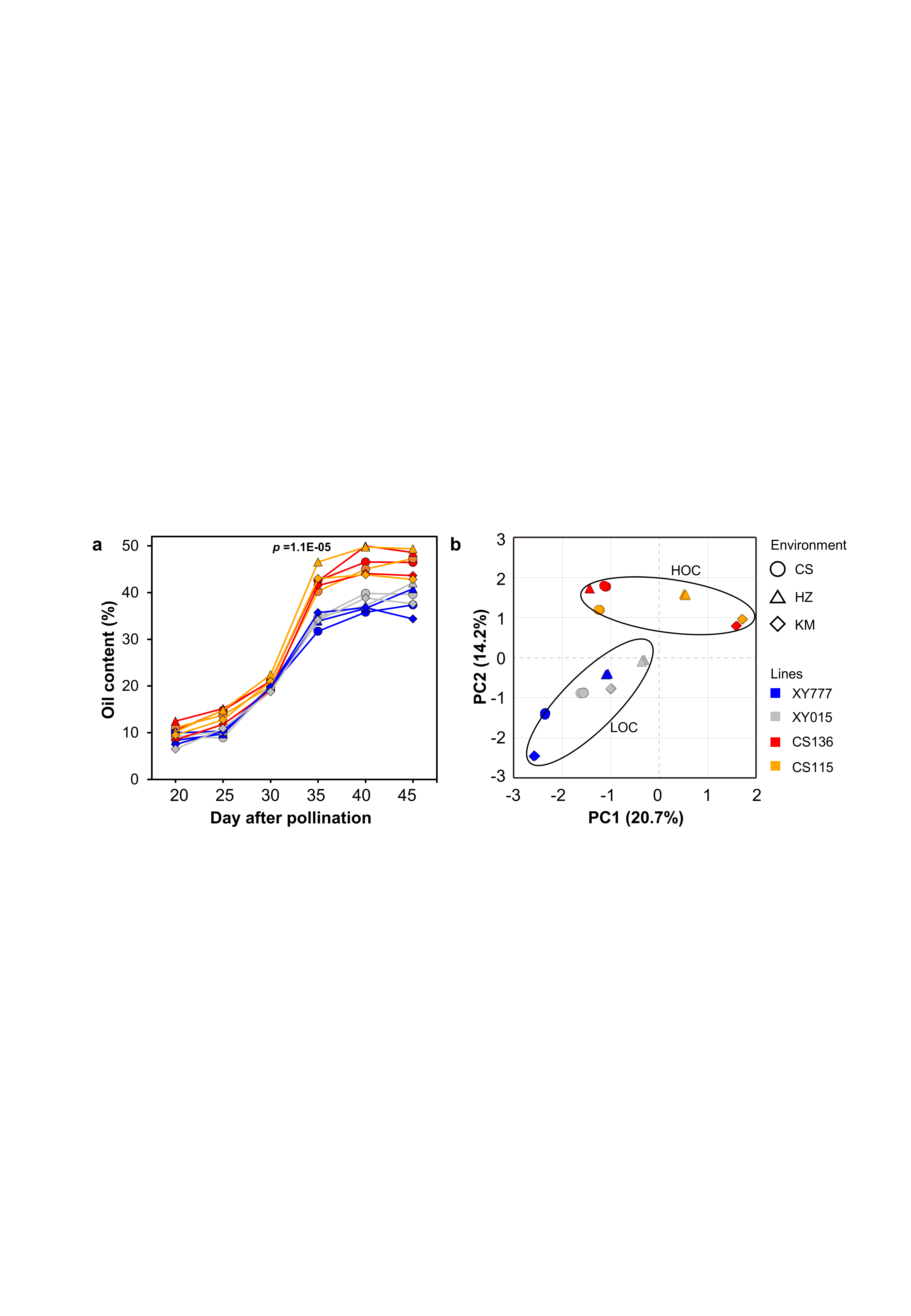

Supplement: Supplementary file 1 — Additional file 1: Figure S1. Oil content phenotype of the development period and PCA distribution for LOC and HOC inbred lines using 35 DAPs FPKM. a The seed oil content of LOC and HOC inbred lines at different environments in different growth stage, the blue and gray, red and orange lines represent LOC and HOC inbred lines, respectively. b PCA distribution for LOC and HOC inbred lines using 35 DAPs FPKM. CS, HZ and KM was signed by cycle, tangle and rhombus, respectively. [file 13068_2024_2480_MOESM1_ESM.tif]

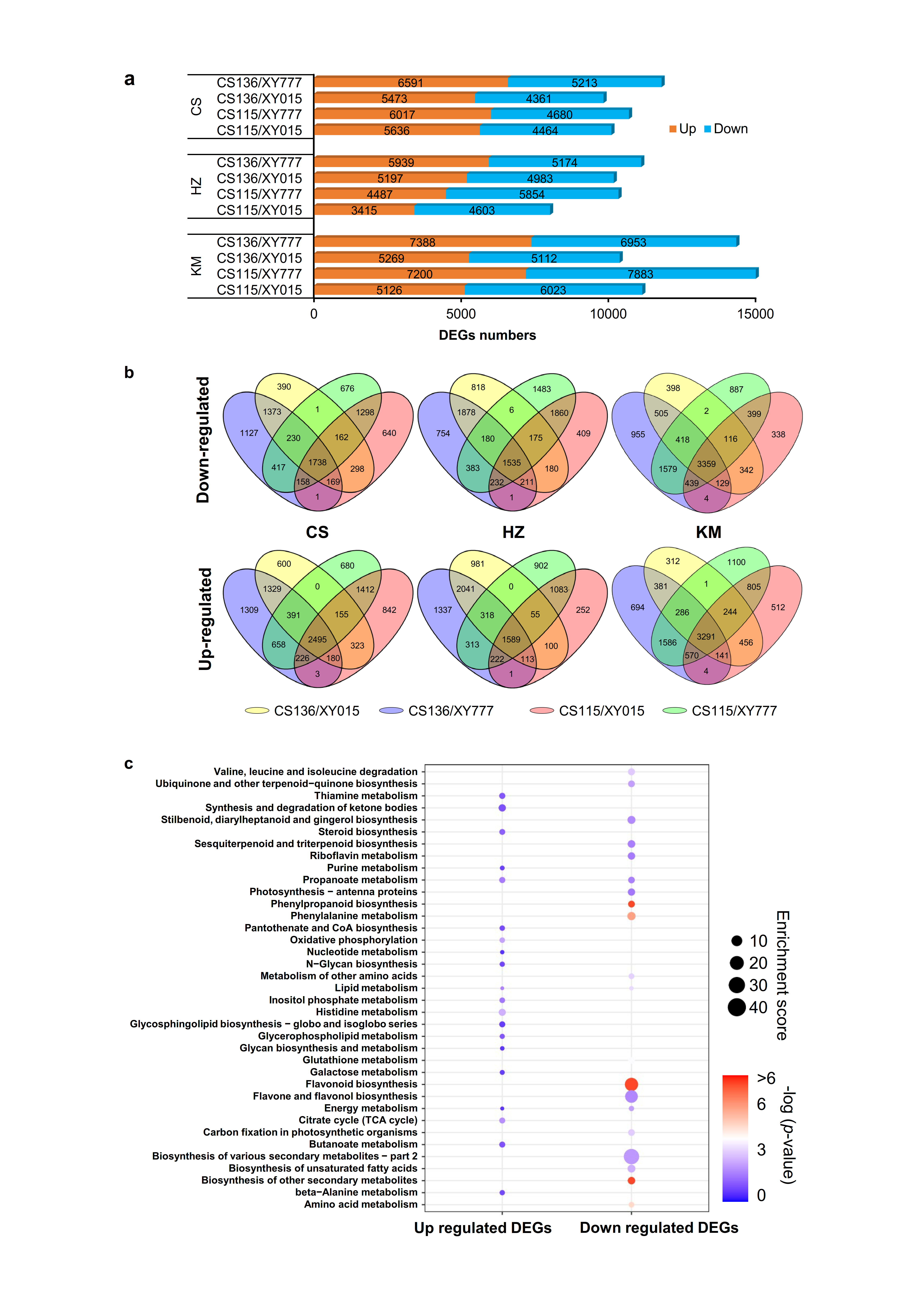

Supplement: Supplementary file 2 — Additional file 2: Figure S2. Overview of DEGs in 35 DAP seeds of the HOC compared to LOC inbred lines. a DEG number of different comparisons at 35 DAP seed. b DEGs overlapped in the same environment under study. c Top 20 KEGG enhancement of common DEGs in three environments. [file 13068_2024_2480_MOESM2_ESM.tif]

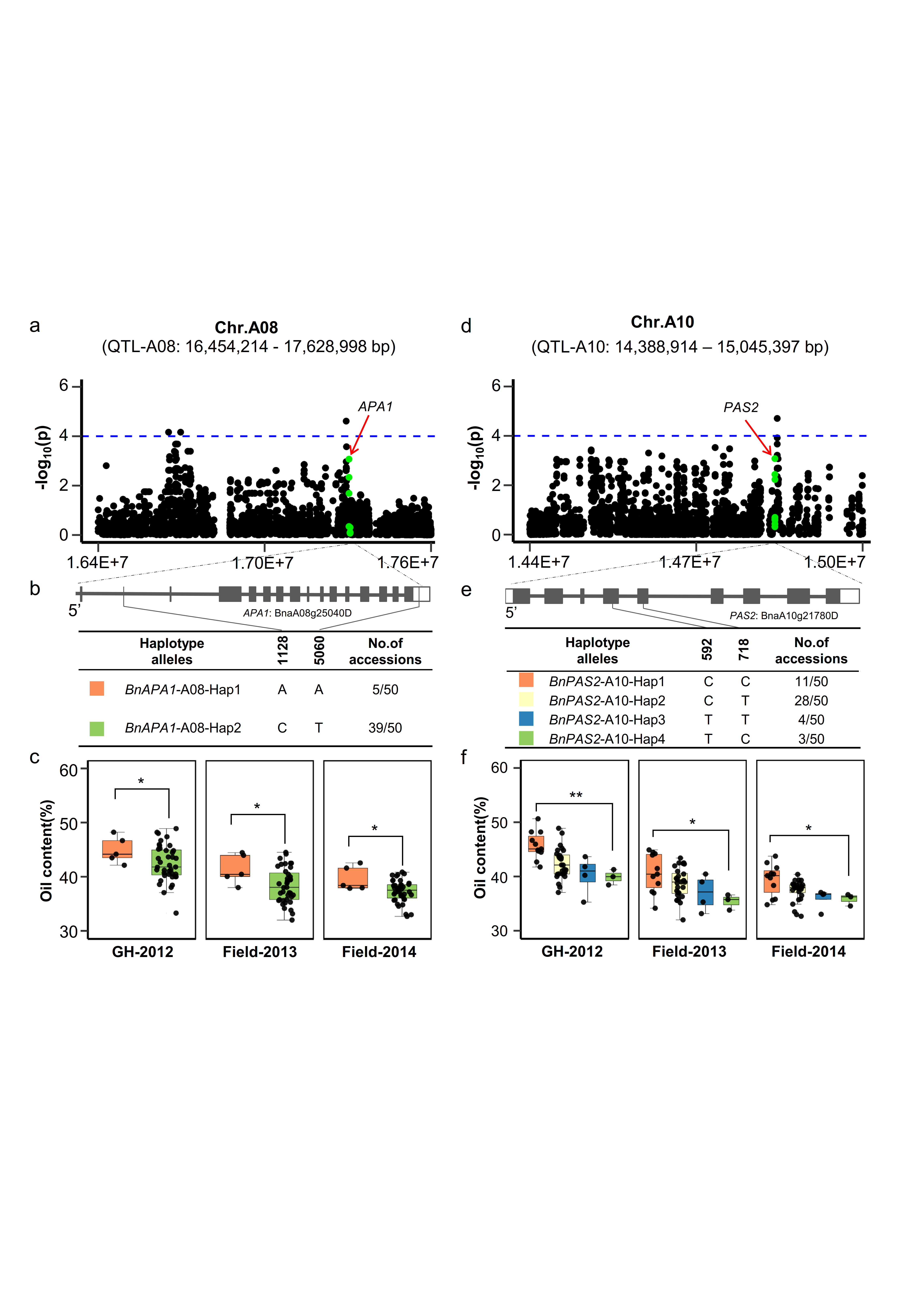

Supplement: Supplementary file 3 — Additional file 3: Figure S3. The analysis of candidate genes in the significant associated QTL-A08 and QTL-A10 regions. Regional Manhattan plot surrounding the peak signals on QTL-A08 (a) and QTL-A10 (d). Green dot indicates the SNPs located in the BnAPA1-A08 (a) and BnPAS2-A10 (d) gene region which are associated with oil content. Genetic structure variations of BnAPA1-A08 (b) and BnPAS2-A10 (e). c and f Boxplots showing comparative analysis between haplotypes related to oil content phenotype. p values show the significance of pairwise comparisons. [file 13068_2024_2480_MOESM3_ESM.tif]

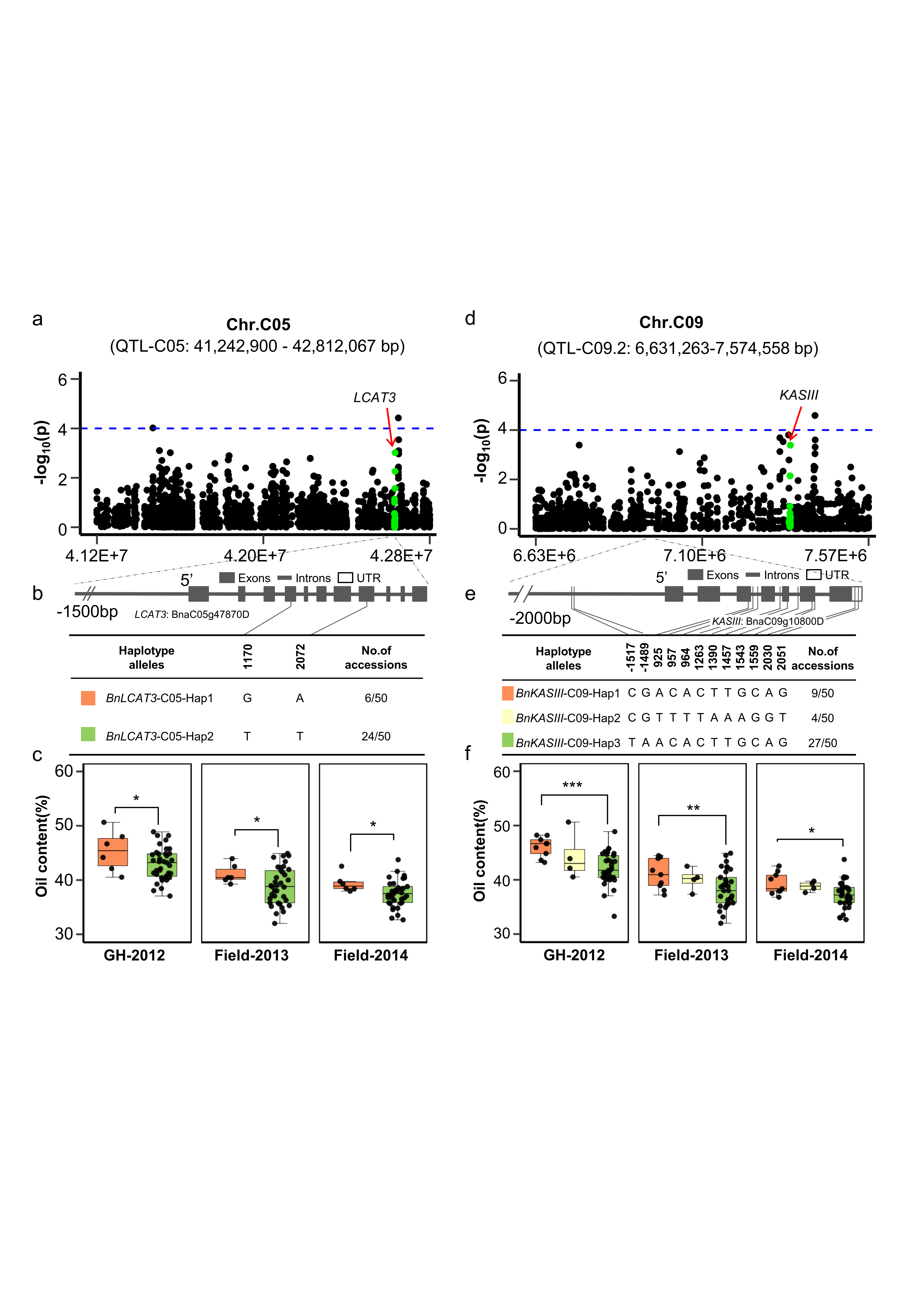

Supplement: Supplementary file 4 — Additional file 4: Figure S4. The analysis of candidate genes in the significant associated QTL-C05 and QTL- QTL-C09.2 regions. Regional Manhattan plot surrounding the peak signals on QTL-C05 (a) and QTL-C09.2 (d). Green dot indicates the SNPs located in BnLCAT3-C05 (a) and BnKASIII-C09 (d) which associated with oil content. Genetic structure variations of BnLCAT3-C05 (b) and BnKASIII-C09 (e), numbers indicate the SNP positions from gene start site. c and f Boxplots showed comparative analysis between haplotypes related to oil content phenotype. p values show the significance of pairwise comparisons. [file 13068_2024_2480_MOESM4_ESM.tif]

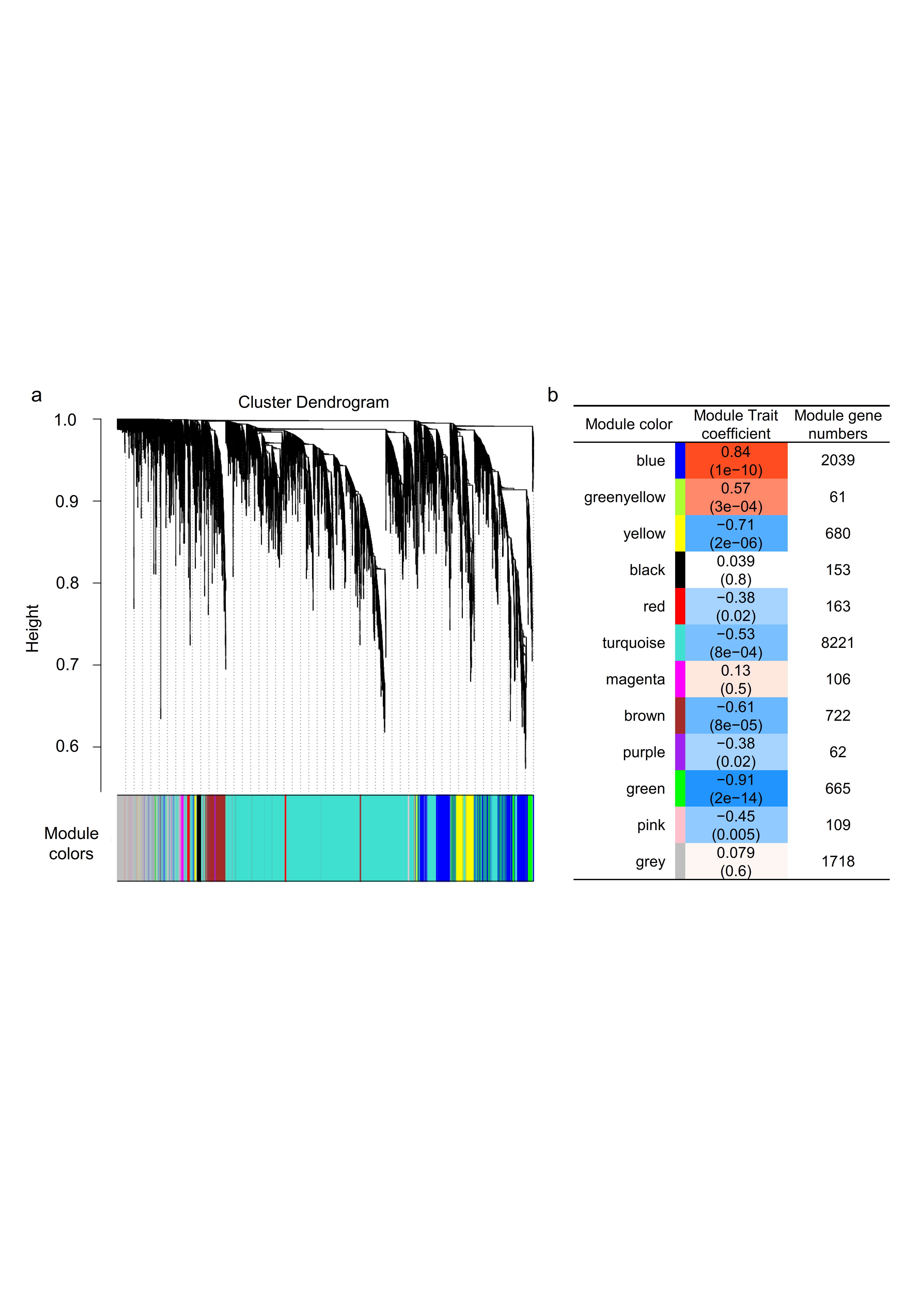

Supplement: Supplementary file 5 — Additional file 5: Figure S5. The result of co-expression network analysis. a Cluster dendrogram of WGCNA gene modules. b The information of module-trait coefficient and module gene numbers. [file 13068_2024_2480_MOESM5_ESM.tif]

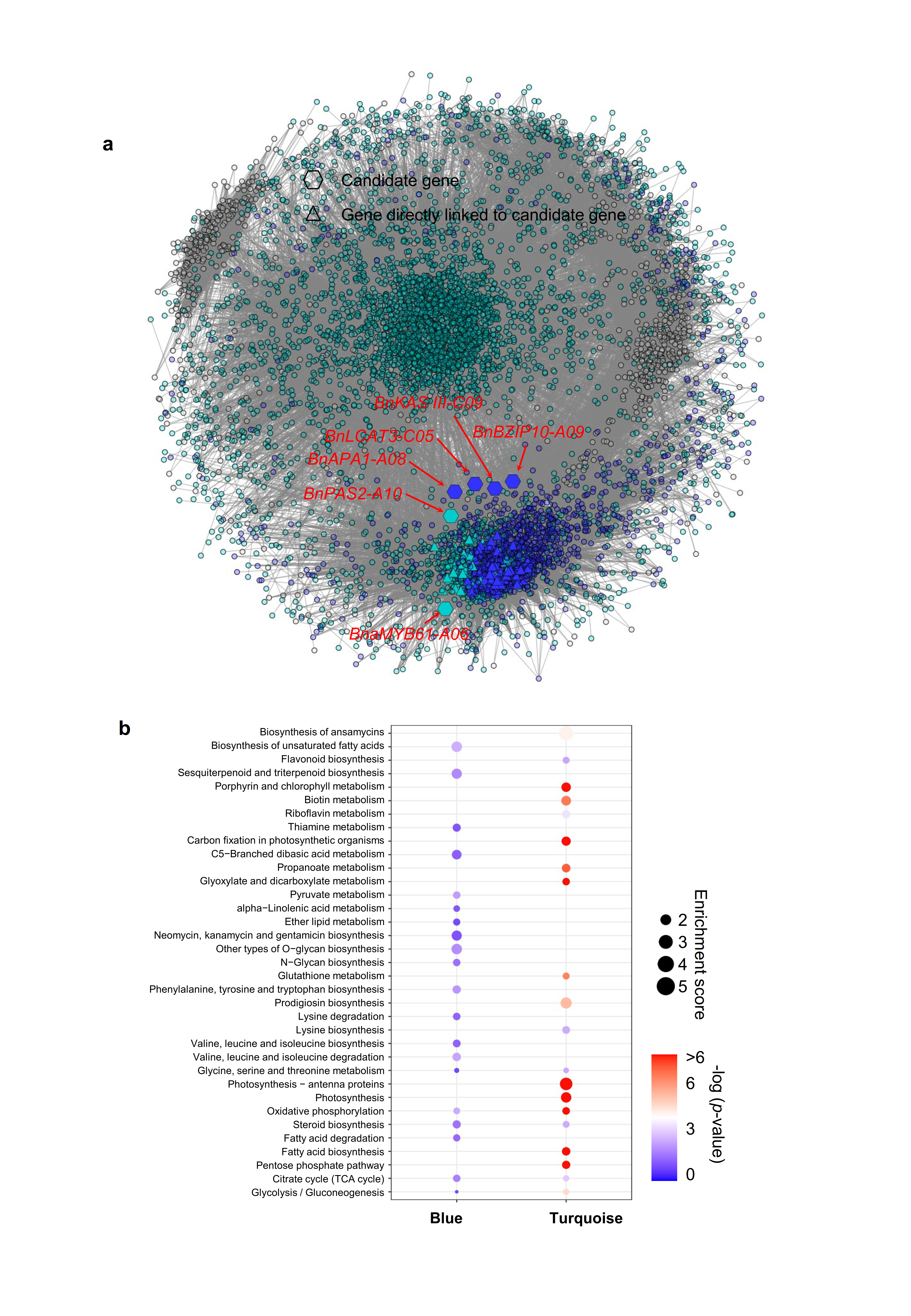

Supplement: Supplementary file 6 — Additional file 6: Figure S6. Co-expression network analysis. a Whole co-expression network exhibit, hexagon nodes represent eight candidate genes, triangle nodes represent genes directly linked to the candidate gene. The blue and turquoise nodes represent blue and turquoise module genes. b Top 20 KEGG enhancement of blue and turquoise module genes. [file 13068_2024_2480_MOESM6_ESM.tif]
